# Tuning High-Voltage Cu–Fe Redox in P2-Type Layered Metal Oxide Cathodes for Sodium-Ion Batteries

**DOI:** 10.1021/acsomega.6c08581

**Published:** 2026-10-01

**Authors:** Hina Parappan, Greeshma Caroline, Shantikumar V. Nair, Senthilkumar Baskar

**Affiliations:** School of Nanosciences and Molecular Medicine, 326614 Amrita Vishwa Vidyapeetham, Kochi, Kerala 682041, India

## Abstract

P2-type layered oxides have gained significant attention in energy storage applications due to their superior rate capability and cycling performance. However, P2-type layered oxides suffer from low sodium content and irreversible phase transitions at high voltages, limiting reversible capacity and long-term durability. Although Cu–Mn-based systems offer a high operating voltage through the Cu^2+^/Cu^3+^ redox couple, the accessible voltage window remains narrow, resulting in restricted capacity. In this work, we employ a compositional engineering strategy by introducing Fe into the Cu–Mn framework to enhance both structural stability and redox activity. Fe incorporation enhances structural stability and redox activity by introducing additional cationic redox centers. The synergistic involvement of the Cu^2+^/Cu^3+^ and Fe^3+^/Fe^4+^ redox couples maintains a high average voltage (∼3.54 V) while improving electrochemical reversibility. The resulting P2–Na_0.7_Cu_0.3_Mn_0.6_Fe_0.1_O_2_ cathode demonstrates a reversible capacity exceeding ∼90 mAh g^–1^ when combined with CNTs, along with excellent cycling stability. Ex situ XRD analysis reveals reversible phase evolution, confirming enhanced structural robustness during cycling. This study highlights the effectiveness of Fe substitution in optimizing Cu–Mn cathode chemistry, offering a promising pathway for high-energy and durable sodium-ion battery systems.

## Graphical abstract

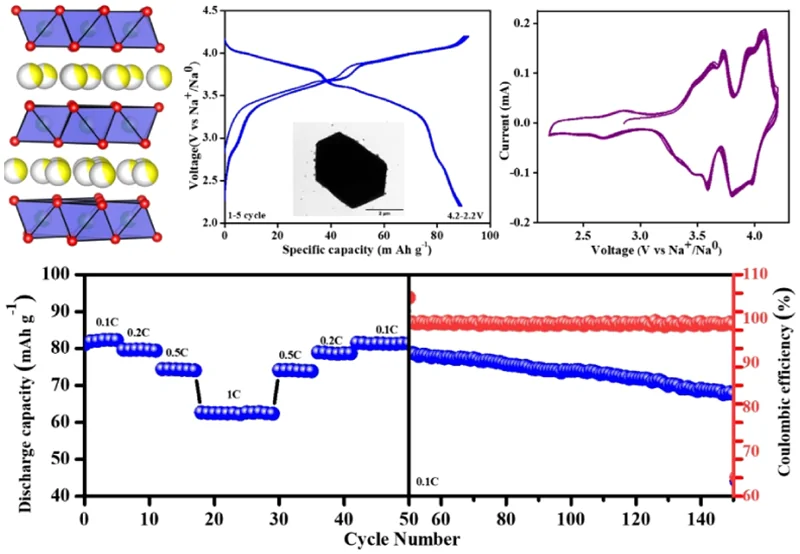

## 1 Introduction

Sodium-ion batteries have become a promising option for cost-effective, large-scale energy storage systems, particularly for renewable energy integration.^1–3^ Among the various components of a battery, the cathode material is of critical importance, as it accounts for 30–40% of the overall cost and significantly influences the energy density of the batteries.^4^ For decades, sodium-based layered oxides (Na_*x*_TMO_2_, where TM denotes transition metals) have attracted significant interest, due to their high specific capacity, environmental compatibility, and ease of synthesis.^5,6^ Among the various structures, P2-type layered oxides have received greater attention due to their high-rate capability, efficient ion diffusion pathways, and elevated average voltage.^2,7,8^ However, these materials are inherently limited by their restricted operating voltage, because when pushed to higher voltages, the P2–O2 phase transition and other structural changes are triggered, leading to a reduced average voltage and compromised long-term cycling stability.^9,10^ Additionally, sodium deficiency at high voltages further accelerates structural instability.^11^

Recently, copper–manganese-based cathode materials have attracted considerable attention due to their promising electrochemical properties, particularly their high average voltage and excellent structural stability.^12–14^ These materials offer a compelling alternative for next-generation sodium-ion batteries, as they balance performance, cost-effectiveness, and environmental sustainability.^15^ Kang et al., demonstrated that in the Cu–Mn system, copper plays a crucial role in improving the electrochemical performance of cathode materials, thereby delivering a high average potential of approximately 3.7 V through the Cu^2+^/Cu^3+^ redox couple.^16^ Furthermore, an increased copper content raises the oxidation state of manganese, stabilizing it in the Mn^4+^ state and effectively suppressing Jahn–Teller distortion. This material exhibits an initial capacity of approximately 80 mAh g^–1^, along with excellent cycling stability. Their study further revealed that while increasing copper content (Cu ≥ 0.33) leads to a higher average voltage, it also promotes the formation of CuO impurities, which can adversely impact capacity retention. Kim et al., reported the successful synthesis of P2-type Na_0.67_Mn_0.67_Cu_0.33_O_2_ material via spray pyrolysis, followed by postsynthetic thermal treatment, yielding phase-pure materials with trace amounts of CuO impurities.^17^ The study identified optimal synthesis conditions, yielding a capacity of 84 mAh g^–1^ with an average voltage of 3.7 V. These results underscore the potential of P2-type NMCO layered oxides as prospective cathode materials for attaining high average voltages in sodium-ion batteries. However, the capacity of this material remains limited due to its constrained voltage range, ultimately restricting its energy density. To mitigate this limitation, our approach seeks to enhance capacity by incorporating additional redox couples while simultaneously stabilizing the structure. This strategy aims to overcome the trade-off between voltage stability and energy density, offering a pathway toward more efficient and high-performance sodium-ion battery cathodes.

Mu et al. developed a high-performance sodium-ion battery prototype using an air-stable, Co/Ni-free O3-type cathode material, O3–Na_0.9_[Cu_0.22_Fe_0.30_Mn_0.48_]­O_2_.^18^ In this system, Cu was doped with Fe to enhance the stability of the Fe-based layered structure. The material demonstrated a reversible capacity of approximately 100 mAh g^–1^ with an initial Coulombic efficiency of 90.4% and an average operating voltage of 3.2 V. The Cu^2+/3+^ and Fe^3+/4+^ redox couples contribute to the 0.4 e^–^ transfer per formula unit, while Cu incorporation also helps mitigate air stability issues. Xu et al., investigated a Na*_x_* (Cu–Fe–Mn)­O_2_ system with varying 3d transition metal compositions, aiming to optimize its electrochemical performance.^19^ Their study found that adjusting the Mn/Fe ratio significantly impacted the redox potentials of the Fe^3+^/Fe^4+^ and Mn^3+^/Mn^4+^ redox couples, likely due to super exchange interactions and the Jahn–Teller effect. The material demonstrated a reversible capacity of 91 mAh g^–1^, an energy density of 252 Wh kg^–1^, and an average operating voltage of 2.76 V. This average voltage is notably lower compared to the reported works. These differences emphasize the need for further exploration to optimize voltage stability and overall performances. From both studies, it is evident that Fe substitution plays a key role in stabilizing the system. Both copper and iron exhibit redox activity, and their synergistic interactions are expected to enhance capacity while reinforcing structural stability. This substitution strategy aims to activate additional cationic redox processes, ultimately improving energy density and cycling stability.^20^ Such advancements are crucial for the development of high-performance sodium-ion battery cathodes.

In this study, Fe was substituted for Cu and Mn in an effort to optimize the stability, capacity, and voltage capability of the cathode. This substitution strategy was designed to promote enhanced cationic redox activity, which, in turn, was expected to increase overall energy density and cycling stability. Fe was selected, considering its ability to stabilize the layered framework and participate in reversible cationic redox reactions alongside Cu and Mn. While Cu^2+^/Cu^3+^ serves as a high-voltage redox couple, Fe^3+^/Fe^4+^ contributes additional redox capacity and enhances structural robustness. The incorporation of Fe also promotes the oxidation of Mn^3+^ to Mn^4+^, thereby mitigating Jahn–Teller distortion and improving lattice reversibility during Na^+^ insertion and extraction. Building on this substitution rationale, the present study systematically investigates Fe-modified Cu–Mn-based P2-layered oxides under controlled sodium-excess conditions to evaluate their structural stability and electrochemical response. Particular emphasis is placed on how compositional tuning influences redox behavior, polarization characteristics, and cycling reversibility within the practical operating voltage range.

## 2 Experimental Method

### 2.1 Preparation of Na_0.7_Cu_0.3_Mn_0.6_Fe_0.1_O_2_ (NCFM)

Sodium-layered oxide cathode material (Na_0.7_Cu_0.3_Mn_0.6_Fe_0.1_O_2_) was synthesized via a citric acid-assisted sol–gel method. Stoichiometric amounts of sodium acetate (C_2_H_3_NaO_2_·3H_2_O, SRL, 99.5%) with 5 wt % (NCFM5) and 10 wt % (NCFM10) excess sodium, copper acetate ((CH_3_COO)_2_Cu·H_2_O, Sigma-Aldrich, ≥98%), manganese acetate (C_4_H_6_MnO_4_·4H_2_O, SRL, ≥99.0%), and ferric nitrate (Fe­(NO_3_)_3_·9H_2_O, SRL, 98%) were dissolved in 40 mL of deionized water, with citric acid (C_6_H_8_O_7_·H_2_O, EMPLURA) used as the chelating agent. The resulting solution was stirred at 80 °C until gelation occurred, after which it was precalcined at 400 °C for 5 h and subsequently calcined at 900 °C for 12 h in air. The Na_0.7_Cu_0.3_Mn_0.6_Fe_0.1_O_2_ sample prepared with the stoichiometric sodium content is denoted as NCFM0. The cathode materials synthesized with 5 and 10 wt % excess sodium precursors are referred to as NCFM5 and NCFM10, respectively.

### 2.2 Materials Characterization

X-ray diffraction (XRD) measurements were conducted using a Bruker D2 diffractometer with a Cu Kα radiation source to analyze the crystal structure of the as-prepared samples. Lattice parameters were subsequently calculated via profile matching using the Match and the FullProf Suite software. Morphological characteristics were examined using a field emission scanning electron microscope (FESEM, JEOL JSM 6490). High-resolution lattice imaging and elemental mapping of the optimal sample were conducted via transmission electron microscopy (TEM, FEI Tecnai G2) coupled with energy-dispersive spectroscopy (EDS). Surface chemical properties were analyzed using X-ray photoelectron spectroscopy (XPS, Axis Ultra, Kratos Analytical, UK). Ex situ XRD was also conducted using the same Bruker D2 diffractometer, employing a Swagelok-type cell. Electrode materials were prepared by thoroughly mixing the active material (160 mg) with carbon black (20 mg, Timcal Super C65) and polyvinylidene fluoride (PVDF, 20 mg, Sigma-Aldrich) for 30 min. A uniform slurry was then formed by adding an appropriate amount of *N*-methyl-2-pyrrolidone (NMP, 99.5%, Sigma-Aldrich). This slurry was coated onto aluminum foil with thickness 35 μm and dried at 80 °C for 15 min. The dried electrodes were compressed and punched into discs with a diameter of 0.95 mm. The electrolyte used was a 1 M solution of sodium hexafluorophosphate (NaPF_6_) dissolved in propylene carbonate (PC) and 5 wt % of fluoroethylene carbonate (FEC) is added. Whatman glass fiber filters were used as separators. Cell was assembled inside an argon-filled glovebox using a Swagelok-type cell. Galvanostatic charge–discharge (GCD) and electrochemical impedance spectroscopy (EIS) were performed using a Biologic BCS805 system within a voltage range of 2.2–4.2 V at 0.1C. Cyclic voltammetry (CV) measurements were conducted using an Autolab (PGSTAT302N) electrochemical workstation.

## 3 Results and Discussion

The as-synthesized compositions (NCFM0, NCFM5, and NCFM10), prepared via the sol–gel method, were characterized for their crystal structure using X-ray diffraction (XRD) as shown in Figure 1a. All samples crystallize in a P2-type layered structure, indexed to the hexagonal *P6*_*3*_/*mmc* space group, typical of sodium-layered transition-metal oxide.^21–23^ All compositions exhibited a minor CuO impurity phase; however, the relative intensity of these peaks diminished with increasing excess sodium content, suggesting improved phase purity at higher Na concentrations.^11^

**Figure 1:**
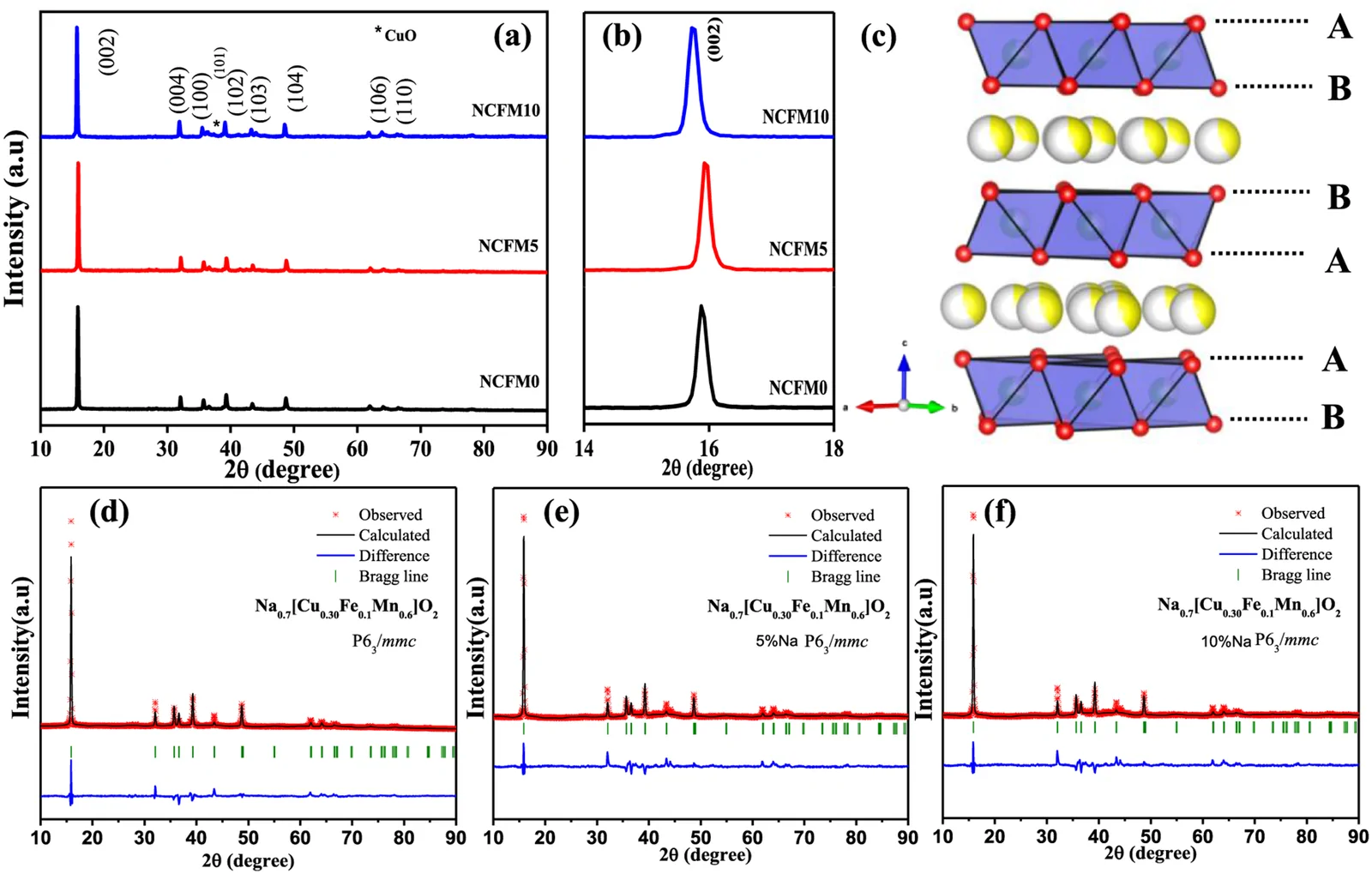
(a) XRD patterns of as-synthesized P2-type cathode materials NCFM0, NCFM5, and NCFM10. (b) Magnified view of the (002) XRD peak, comparing pristine and excess Na-added samples. (c) Schematic illustration of the crystal structure of P2-type layered oxide. (d–f) Profile-matched XRD patterns of NCFM0, NCFM5, and NCFM10.

The magnified XRD profile of the (002) reflection reveals distinct peak shifts among the samples (Figure 1b). Compared to NCFM0, the NCFM5 composition exhibits a slight shift toward higher 2θ values, indicating a contraction along the *c*-axis. This is likely due to partial occupation of interstitial or vacant Na sites, resulting in stronger electrostatic interactions and shortened Na–O bond lengths, thereby reducing the interlayer spacing.^24^ In contrast, the (002) peak for NCFM10 shifts to a lower angle, indicating lattice expansion. This expansion is attributed to excess sodium content, which induces structural disorder and interlayer repulsion.^25^ The optimal balance between structural expansion and Na-ion transport at 10 wt % excess Na is likely controlled by the synergistic effects of Fe and Cu substitution, which induce structural disorder while facilitating ion mobility.^11,26,27^ The crystal structure illustration of the P2-type layered oxide, presented in Figure 1c, depicts the characteristic ABBA oxygen stacking sequence, in which Na^+^ ions reside in both edge- and face-sharing prismatic sites within the interlayer spaces.^23^ Profile matching was performed for all compositions, as shown in Figure 1d–f, to evaluate structural parameters. The calculated *a*/*c* ratios and unit cell volumes for NCFM0, NCFM5, and NCFM10 are presented in Table S1.

Field-emission scanning electron microscopy (FESEM) (Figure 2a–c) was employed to investigate the surface morphology of the synthesized samples (NCFM0, NCFM5, and NCFM10). FESEM images reveal well-defined particle morphologies. Energy-dispersive X-ray spectroscopy (EDS) elemental mapping for NCFM0, NCFM5, and NCFM10, given in Figures S1–S3, confirms a homogeneous elemental distribution, verifying the uniform incorporation of all constituent elements.^21,22^ Transmission electron microscopy (TEM) was conducted to gain deeper insights into the microstructural features of NCFM10, revealing a crystalline structure with a distinct hexagonal morphology, as shown in Figure 2d. High-resolution transmission electron microscopy (HR-TEM) (Figure 2e) estimated an interplanar spacing of approximately 0.27 nm, which corresponds to the (002) plane of the P2-type layered structure. Selected area electron diffraction (SAED) patterns (Figure 2f) confirmed the crystalline nature of the materials and showed well-resolved hexagonal diffraction spots, with measured *d*-spacing values close to 0.267 nm, further corresponding to the (002) reflection. Elemental distribution mapping via energy-dispersive X-ray spectroscopy (EDS) confirmed the uniform distribution of all constituent elements (Na, Cu, Fe, Mn, and O), indicating successful incorporation and homogeneity across the samples (Figure 2g).^21,28,29^

**Figure 2:**
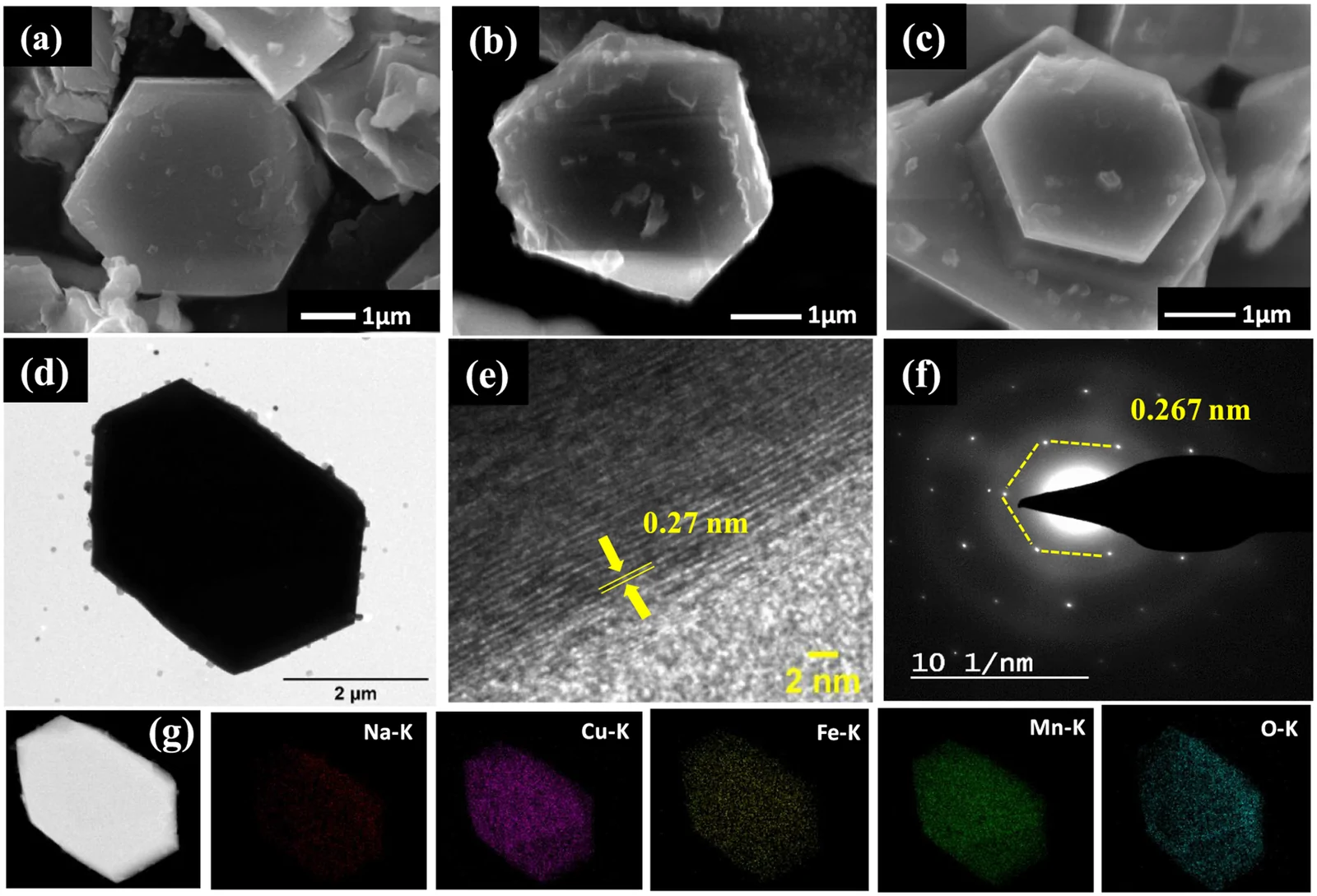
Morphological characterizations of NCFM samples: (a–c) FESEM images of NCFM0, NCFM5, and NCFM10, respectively; (d, e) TEM and high-resolution TEM (HRTEM) images of NCFM10; (f) selected-area electron diffraction (SAED) pattern of NCFM10; and (g) elemental mapping of NCFM10.

X-ray photoelectron spectroscopy (XPS) was employed to probe the surface chemistry, elemental composition, oxidation states, and potential surface impurities of NCFM0, NCFM5, and NCFM10. The Na 1s spectra (Figure S4a) confirm the presence of sodium, exhibiting a characteristic peak at ∼1072 eV for NCFM5 and NCFM10, consistent with Na^+^ species in the structure. The progressive increase in peak intensity from NCFM5 to NCFM10 indicates a higher surface sodium concentration with increasing Na content. In the Cu 2p region (Figure 3a), distinct peaks at ∼933 eV (Cu 2p_3/2_) and ∼953 eV (Cu 2p_1/2_), together with pronounced shakeup satellites, verify the existence of Cu^2+^ in the oxide framework.^30^ The Fe 2p spectra (Figure S4b) exhibit distinct peaks at ∼710–720 eV (Fe 2p_3/2_) and ∼720–730 eV (Fe 2p_1/2_), characteristic of Fe^3+^ in an oxide environment.^31^ The presence of satellite peaks, typically associated with charge transfer effects, further confirms the +3 oxidation state of iron. The Mn 2p spectra in Figure 3b display a Mn 2p_3/2_ peak at ∼642–644 eV and a Mn 2p_1/2_ broader feature at ∼653–655 eV, consistent with the coexistence of Mn^3+^/Mn^4+^ in the oxide framework.^32^ A more pronounced low-energy component from NCFM5 to NCFM10 suggests that the incorporation of 10 wt % excess sodium increases the Mn^3+^ content to satisfy charge balance requirements. The observed satellite features arise from multiplet splitting and shake-up processes, characteristic of manganese in mixed oxidation states. The O 1s spectra (Figure 3c) exhibit a primary peak at ∼529–530 eV, characteristic of lattice oxygen in metal oxides. A secondary peak at ∼531 eV is attributed to surface hydroxyl (−OH) species.^33^ The C 1s peak at 284.7 eV was used as the reference for binding energy calibration (Figure S4c).

**Figure 3:**
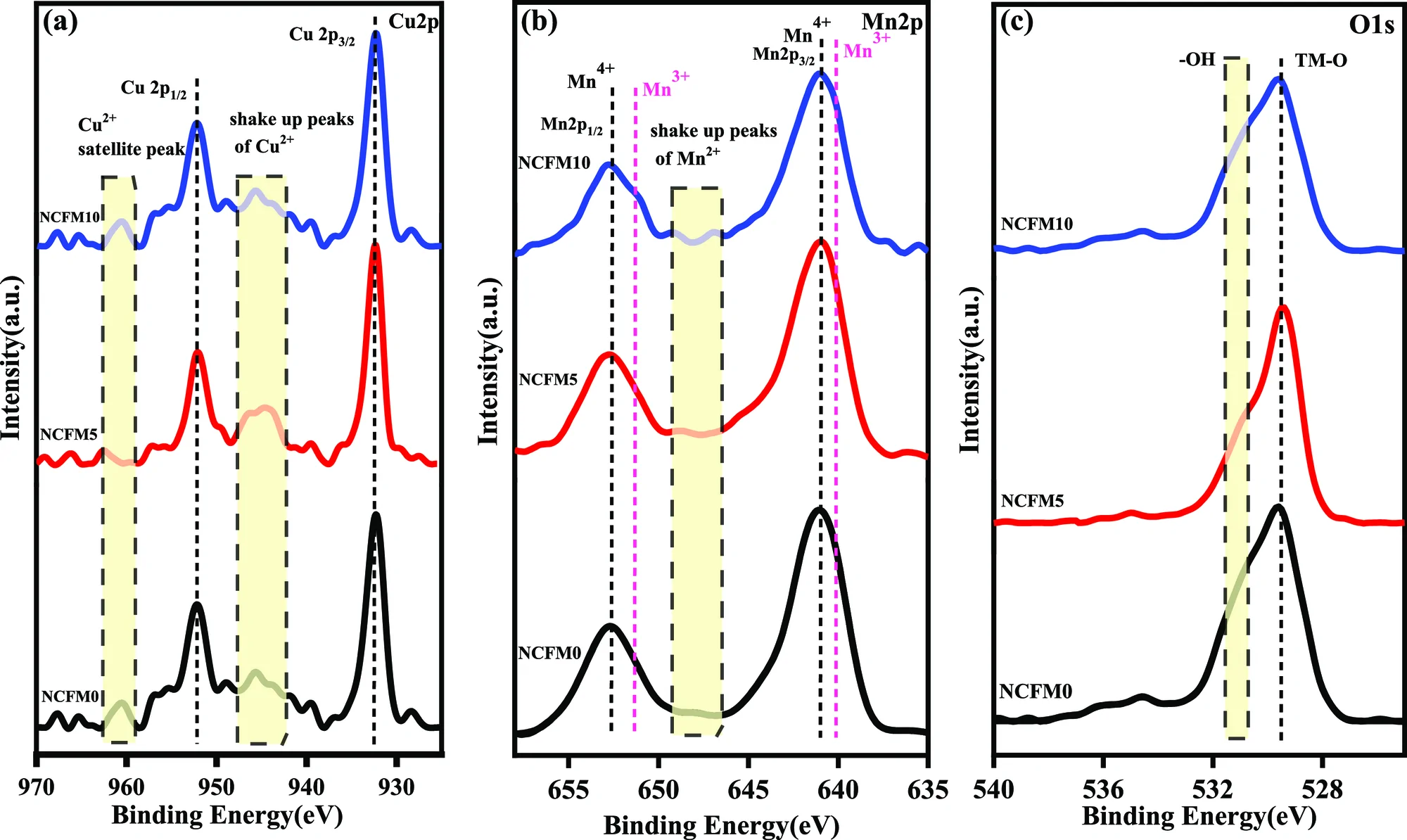
X-ray photoelectron spectroscopy (XPS) spectra of the materials NCFM0, NCFM5, and NCFM10, illustrating the elemental composition and chemical states: (a) Cu 2p spectrum, (b) Mn 2p spectrum, (c) O 1s spectrum.

The electrochemical performance of the as-prepared cathode materials was systematically evaluated over the initial five cycles using galvanostatic charge–discharge (GCD) testing in the voltage window of 2.2–4.2 V vs Na^+^/Na^0^ at a current rate corresponding to 0.1C, as shown in Figure 4a–b. NCFM0 delivered an initial discharge capacity of 70 mAh g^–1^, whereas NCFM5 and NCFM10 exhibited higher capacities of 85 and 88 mAh g^–1^, respectively, with average operating voltages of around 3.6 V. As shown in Figure 4a, the initial discharge capacity of NCFM0 is notably lower than that of the second cycle, increasing from 70 to 80 mAh g^–1^. This phenomenon is attributed to sodium deficiency in the material, a common characteristic of Na-deficient cathodes, which often exhibit capacity enhancement after the first cycle.^34–36^ Such behavior arises from the delayed electrochemical activation of previously inactive redox sites and improved Na^+^ accessibility following initial structural relaxation.^35,37^

**Figure 4:**
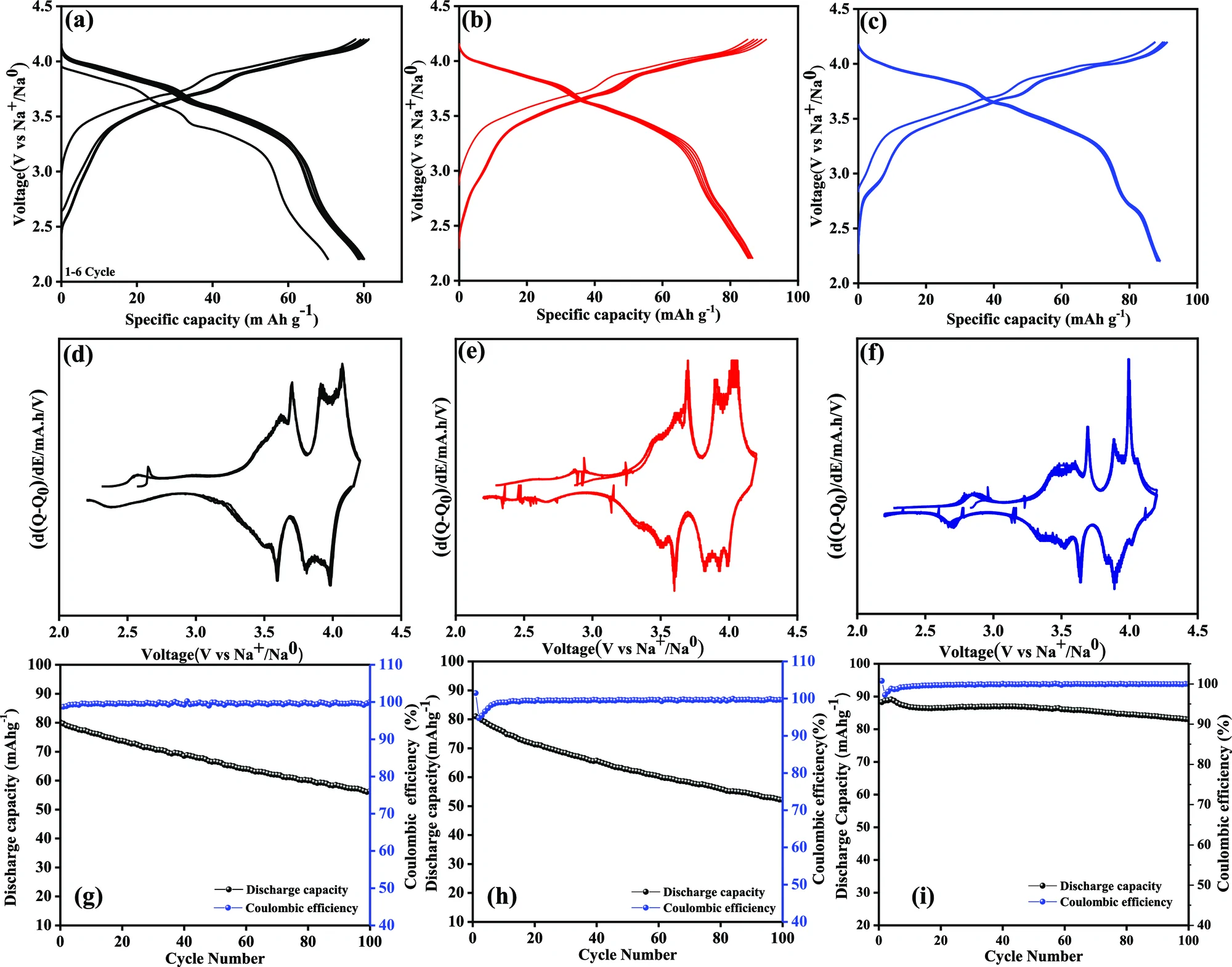
(a–c) Galvanostatic charge–discharge profiles of NCFM0, NCFM5, and NCFM10 samples at 0.1C over the voltage range 4.2–2.2 V. (d–f) Differential capacity (d*Q*/d*V*) profiles within the same voltage window. (g–i) Cycling performance at 0.1C of NCFM0, NCFM5, and NCFM10, respectively.

As shown in Figure 4b, the introduction of 5 wt % excess sodium in NCFM5 effectively compensates for Na deficiency, promoting the formation of additional Mn^3+^ to satisfy charge balance. Upon further increasing the sodium content to 10 wt % in NCFM10 (Figure 4c), a marked improvement in diffusion kinetics is observed, which contributes to superior capacity and rate characteristics.^35,38^ Compared to NCFM0 and NCFM5, NCFM10 exhibits the emergence of a minor plateau in the charge–discharge profile, indicative of additional redox activity. This feature is likely associated with the onset of Na^+^ vacancy ordering and a partial Mn^4+^ → Mn^3+^ reduction, pointing to subtle electronic and structural rearrangements.^39^ The enhanced electrochemical performance of NCFM10 is attributed to the optimized balance between lattice expansion and improved Na^+^ transport, likely enabled by the synergistic effects of Fe and Cu substitution, which promote a more favorable redox environment and facilitate faster ion diffusion.^40–42^ The corresponding differential capacity (d*Q*/d*V*) plots (Figure 4d–f) provide further insights into the redox behavior and kinetic characteristics of the NCFM series. In NCFM0 (Figure 4d), broad and widely separated peaks are observed, indicative of sluggish reaction kinetics and significant polarization. In contrast, NCFM5 (Figure 4e) exhibits more distinct redox features, with well-defined peaks corresponding to Cu and Mn redox processes and a noticeable reduction in polarization relative to NCFM0. NCFM10 (Figure 4f) exhibits a more refined redox activity, retaining pronounced Cu and Fe redox features with only minor Mn contributions, accompanied by a significant reduction in polarization. Although a fraction of Mn^3+^ remains, its optimized level prevents structural instability, thereby enabling a more symmetric and stable redox process. Collectively, the d*Q*/d*V* profiles highlight NCFM10 as the most electrochemically optimized composition, where minimized polarization and balanced redox activity are achieved through fine-tuning of sodium content and transition metal substitution.^43^

The cycling stability of Na_0.7_Cu_0.3_Fe_0.1_Mn_0.6_O_2_ compositions, as depicted in (Figure 4g–i), offers critical insight into their long-term electrochemical durability. NCFM0 (Figure 4g) exhibits relatively stable cycling behavior but with lower capacity retention of approximately 70% after 100 cycles. In contrast, NCFM5 (Figure 4h) delivers a higher initial capacity; however, it undergoes more pronounced capacity fading, retaining only ∼64% after 100 cycles. This decline is likely attributed to residual Na deficiency, even with 5 wt % excess sodium, not all Na in the structure is electrochemically extractable, which may lead to partial structural degradation over extended cycling. Notably, NCFM10 (Figure 4i) demonstrates superior cycling stability, maintaining ∼94% of its initial capacity after 100 cycles and achieving a Coulombic efficiency nearing 99.9%. This excellent stability suggests enhanced structural stability and redox reversibility, likely enabled by the optimized sodium content and synergistic effects of Fe and Cu substitution.^40–42^

To enhance the intrinsic electronic conductivity of the NCFM cathode material, carbon nanotubes (CNTs) were used as conductive additives in the slurry preparation, replacing traditional carbon black. The CNTs form an interconnected network that enhances electron transport, lowers internal resistance, and boosts overall electrochemical performance.^34,40^ The first five cycles of CNT-Na_0.7_Cu_0.3_Fe_0.1_Mn_0.6_O_2_ samples revealed distinct electrochemical profiles across the Na-deficiency gradient (NCFM0 → NCFM10), highlighting the relationship between Na^+^ diffusion, electronic conductivity, and phase transition behavior. Compared to carbon black (CB) additive samples, CNT-NCFM0 (Figure S5a) exhibited overlapping charge–discharge curves with minimal hysteresis, indicating high reversibility and solid-solution-type Na^+^ intercalation, which is superior to the broader hysteresis observed in CB samples. The smooth voltage profile suggests uniform Na^+^ percolation and minimal internal strain, consistent with enhanced structural stability.^40–42^ In NCFM5 (Figure S5d), voltage plateaus near 3.0–3.1 V indicate phase transitions involving Fe^3+^/Fe^4+^ and Mn^3+^/Mn^4+^ redox couples. The polarization is significantly reduced compared to CB, indicating that the conductive CNT network mitigates kinetic hindrances associated with Na vacancy-induced diffusion barriers.^38,39^ In NCFM10 (Figure 5a), the smoother GCD curve with enhanced charge–discharge overlap reflects improved reversibility and reduced polarization, outperforming the CB counterparts. The balanced Na-deficiency level in combination with the CNT matrix enhances structural openness and ion transport efficiency. The CNT–electrode porous matrix facilitates electrolyte penetration and shortens the effective Na^+^ transport distance, thereby promoting more uniform active-material utilization and reducing kinetic limitations.

**Figure 5:**
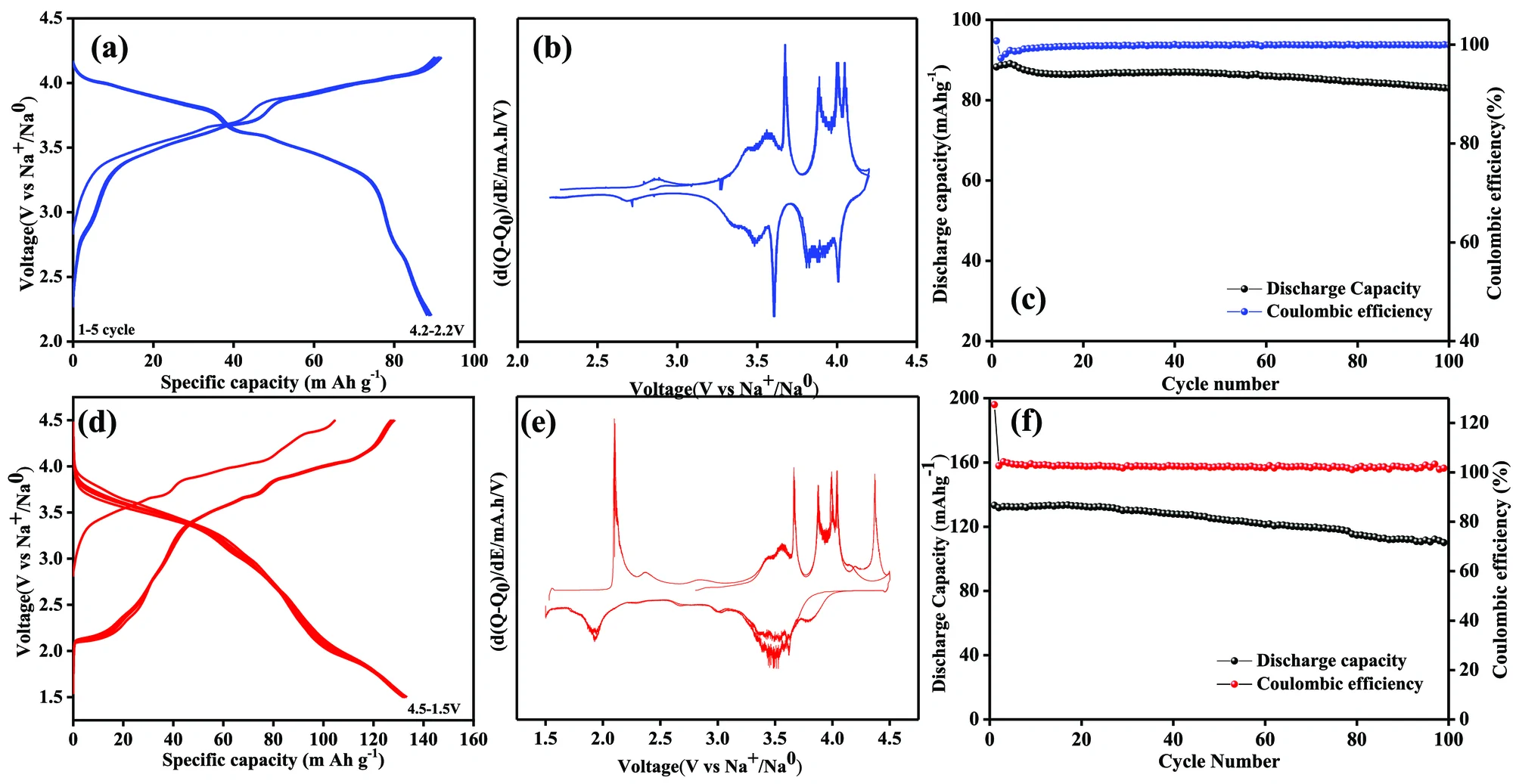
(a) Galvanostatic charge–discharge profiles of CNT additive of NCFM10 samples at 0.1C over the voltage range 4.2–2.2 V. (b) The corresponding differential capacity (d*Q*/d*V*) profiles within the same voltage window. (c) Cycling performance at 0.1C. (d) Galvanostatic charge–discharge profiles of CNT additive of NCFM10 samples at 0.05 C over the voltage range 4.5–1.5 V. (e) The corresponding differential capacity (d*Q*/d*V*) profiles within the same voltage window. (f) Cycling performance at 0.05 C.

Differential capacity (d*Q*/d*V*) plots provided insight into redox mechanisms. For NCFM0-CNT (Figure S5b), the broad anodic (3.2–3.4 V) and cathodic (3.0–3.2 V) peaks signify diffusion-controlled Na^+^ intercalation with minimal structural disruption, more stable than the diffuse and separated peaks observed in CB samples. NCFM5 (Figure S5e) displayed sharper redox peaks (∼3.3 V anodic and ∼3.1 V cathodic), characteristic of Fe and Mn redox activity, yet with reduced polarization compared to CB samples. In NCFM10 (Figure 5b) the broader, symmetric redox peaks (∼3.2 V anodic, ∼3.0 V cathodic) indicate a more reversible intercalation process, highlighting the improved Na^+^ transport environment provided by CNTs.

Cycling performance demonstrated the durability of Na_0.7_Cu_0.3_Fe_0.1_Mn_0.6_O_2_ compositions. Compared to CB-coated variants, NCFM0-CNT (Figure S5c) delivered an initial discharge capacity of ∼84 mAh g^–1^, retaining ∼91% after 70 cycles, showing better retention and higher Coulombic efficiency. NCFM5 (Figure S5f) exhibited slightly higher capacity (∼85 mAh g^–1^) with ∼99.9% retention after 100 cycles, outperforming CB-coated counterparts. NCFM10 (Figure 5c) showed the highest capacity (∼90 mAh g^–1^) with ∼94% retention after 100 cycles at 0.1C within the voltage window of 2.2–4.2 V and nearly 100% Coulombic efficiency, demonstrating superior cycling stability and electrochemical reversibility compared to CB additives.

NCFM10-CNT showed a clear advantage over CB additives due to its high capacity, robust cycling retention, and reversible Na^+^ intercalation. The electrochemical performance of NCFM10 was further evaluated at a current rate of 0.05C across an extended voltage window of 1.5–4.5 V to investigate its behavior under high-voltage operation and deeper Na^+^ (de)­intercalation. When carbon black was used as an additive, NCFM10 exhibited relatively smooth charge–discharge curves with modest hysteresis and partial overlap between the charge and discharge branches (Figure S5g) indicative of moderate polarization and reasonable reversibility. The curve displayed well-defined voltage plateaus, especially around 2.3 and 3.7 V, corresponding to distinct redox processes likely involving Fe^3+^/Fe^4+^ and Mn^3+^/Mn^4+^ transitions. The initial discharge capacity reached ∼125 mAh g^–1^, with an average operating voltage of ∼3.2 V. The differential capacity (d*Q*/d*V*) profile in Figure S5h revealed sharp and discrete peaks in the 2.0–3.0 V and 3.5–4.0 V regions, highlighting multistep phase transitions that, while enhancing redox accessibility, may also induce structural strain and lead to capacity fading over prolonged cycling. The corresponding cycling performance (Figure S5i) confirmed this trend, showing a slight decrease in capacity over six cycles with ∼125 mAh g^–1^ retained and a Coulombic efficiency of ∼99%, reflecting moderate reversibility but potential structural degradation due to repetitive phase changes. In contrast, the NCFM10-CNT sample demonstrated superior electrochemical behavior. The charge–discharge curves (Figure 5d) of NCFM10 at extended window featured minimal voltage hysteresis and near-complete overlap between charge and discharge profiles, indicating reduced polarization and enhanced kinetic reversibility. This enhancement is attributed to the high electronic conductivity and flexible fibrous network of CNTs, which facilitate efficient electron transport and structural accommodation during cycling. The CNT-additive electrode delivered a higher initial discharge capacity of ∼132 mAh g^–1^, with an elevated average voltage of ∼3.3 V. The extended voltage range allowed additional Na^+^ extraction/insertion processes to be accessed, particularly those occurring below 2.2 V and above 4.2 V, thereby contributing to the overall discharge capacity. Also, the lower current rate at 0.05C provides more time for Na^+^ diffusion and electrode reaction kinetics, enabling complete utilization of the electrochemically active material. Additionally, below 2.2 V, there is active participation of Mn redox contributing toward charge compensation, which leads to a capacity increase.

The associated d*Q*/d*V* plot (Figure 5e) showed broader and less intense peaks spanning the same voltage intervals, suggesting a predominantly solid-solution reaction mechanism. This behavior minimizes lattice strain and mechanical fatigue, thereby supporting stable long-term cycling. The cycling performance (Figure 5f) confirmed the improved durability, with the capacity maintained at ∼132 mAh g^–1^ after 10 cycles and a stable Coulombic efficiency of ∼98%, indicative of high reversibility and low parasitic loss. Collectively, the CNT additive significantly enhances both the electrochemical kinetics and cycling stability of NCFM10 by forming a continuous and conductive 3D network that mitigates polarization, buffers structural stress, and supports sustained Na^+^ (de)­intercalation over wide voltage ranges. These results underscore the critical role of conductive matrix architecture in optimizing high-voltage sodium-ion battery cathodes.

Cyclic voltammetry (CV) of NCFM10 was conducted in the voltage range of 4.2–2.2 V at a scan rate of 0.01 mV s^–1^, as shown in Figure 6a. The CV profile displayed a broad anodic region between 3.5 and 4.0 V, with a shoulder near 3.7–3.8 V, associated with the Cu^2+^/Cu^3+^ redox couple during Na^+^ deintercalation.^43,44^ The broadness of this region suggests sluggish redox kinetics or overlapping electrochemical processes within the mixed transition-metal matrix. During the cathodic sweep, a distinct reduction peak was observed between 2.5 and 3.0 V, with a notable feature at ∼2.7–2.8 V, corresponding to Mn^3+^/Mn^4+^ and Fe^3+^/Fe^4+^ transitions. Additionally, several minor cathodic features between 3.0 and 3.5 V were observed, likely arising from Fe^3+^/Fe^4+^ activity modulated by Cu incorporation. The broad and distributed nature of these redox features reflects the complex but reversible multielectron reactions typical of layered transition-metal oxides in sodium-ion systems, favoring enhanced specific capacity while maintaining electrochemical reversibility.

**Figure 6:**
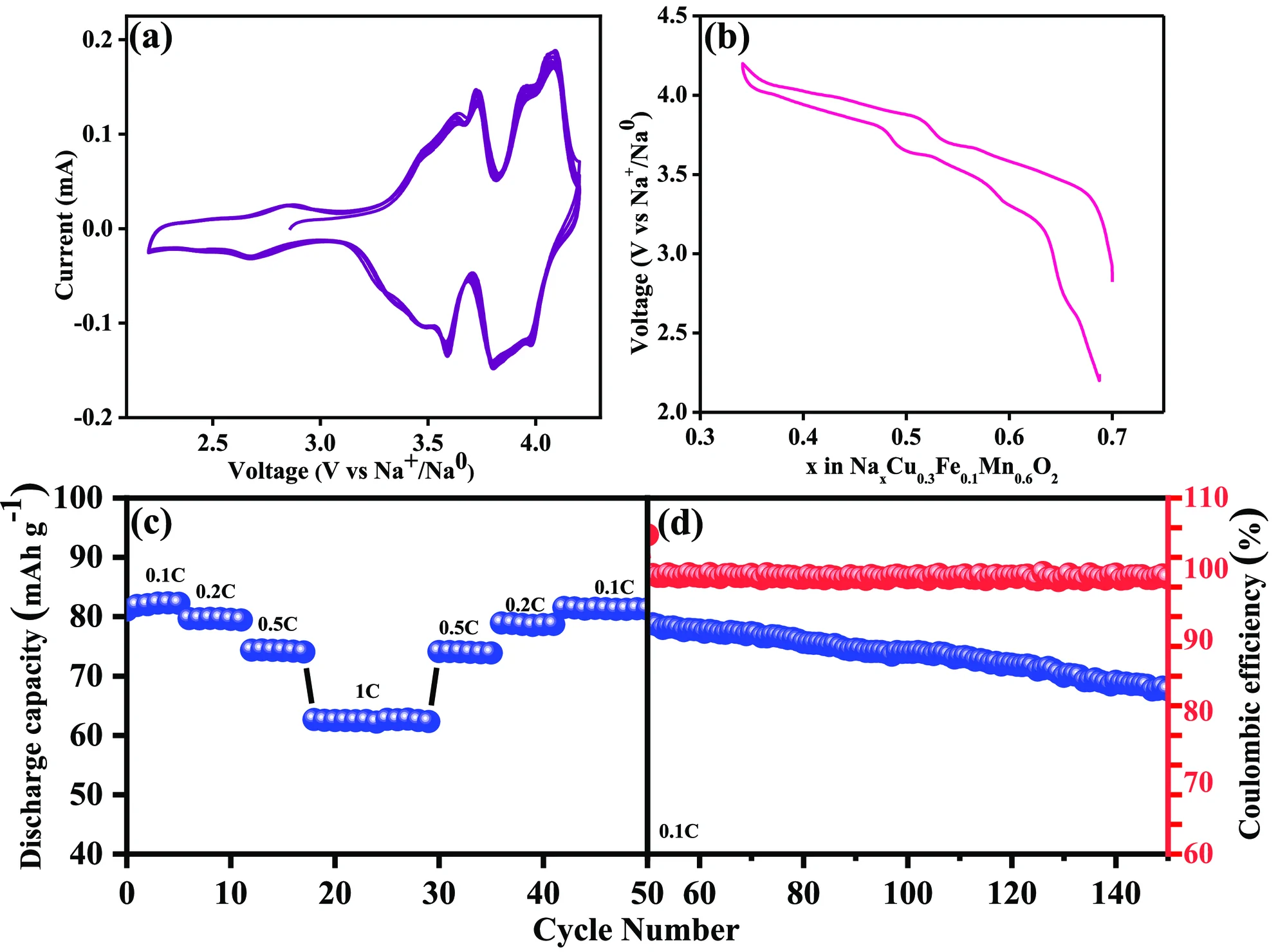
(a) Cyclic voltammetry profile measured in the voltage range of 2.2–4.2 V. (b) Number of electrons transferred (x) as a function of potential. (c) Rate capability study of NCFM10 at different current rates (0.1, 0.2, 0.5, 1, and 2 C). (d) Cyclic performance at a current rate of 0.1C.

To further understand the sodium intercalation behavior, the plot of the number of electrons transferred (*x*) versus potential (Figure 6b) was analyzed within the range of *x* = 0.7 to 0.3, mapping the evolution of the sodium content during (de)­sodiation. Here, *x* = 0.7 denotes a partially desodiated state at the onset, whereas *x* = 0.3 represents a significantly desodiated or subsequently resodiated state. This variation demonstrates efficient Na^+^ intercalation/deintercalation processes and indicates a stable structural framework that supports continuous sodium-ion movement without significant degradation.^45^

Subsequently, the rate capability of NCFM10 was systematically assessed by subjecting the electrode in different current densities from 0.1C to 1C, as depicted in Figure 6c. At the rate of 0.1C, NCFM10 delivered a stable discharge capacity of ∼82 mAh g^–1^, corresponding to 100% retention, indicative of efficient Na^+^ transport and robust redox activity under low-current conditions. Upon increasing the rate to 0.2C, a slight decrease in capacity to ∼78 mAh g^–1^ was observed (97.5% retention), attributable to increased polarization and moderate diffusion resistance. Further elevating the rate to 0.5C resulted in a discharge capacity of ∼72 mAh g^–1^ (96% retention relative to 0.2C), reflecting moderate kinetic limitations due to faster ion transport demands. At 1C, the capacity declined more notably to ∼65 mAh g^–1^, corresponding to 81% retention relative to the initial 0.1C value, suggesting significant Na^+^ diffusion constraints and enhanced charge transfer resistance at higher current rates.^28^ Remarkably, when the current rate was reverted to 0.1C, the capacity largely recovered, indicating that the rate-induced capacity loss was predominantly reversible and related to transient polarization rather than irreversible structural degradation. Following the rate capability evaluation, NCFM10 was subjected to prolonged cycling at 0.1C for 100 cycles in Figure 6d. The material retained ∼85% of its initial capacity, demonstrating excellent long-term cycling stability. Minor capacity decay observed after 50 cycles, despite the constant current rate, can be attributed to cumulative structural fatigue, gradual electrolyte decomposition, and slight polarization buildup. Overall, the enhanced electrochemical performance of NCFM10 can be associated with the combined contribution of Cu- and Fe-centered redox processes within a structurally robust TM-O framework. The feasibility of such coupled redox chemistry is well established in closely related P2 Cu–Fe–Mn oxides. In P2–Na_7/9_Cu_2/9_Fe_1/9_Mn_2/3_O_2_, Li et al. identified Cu^2+^/Cu^3+^and Fe^3+^/Fe^4+^ redox through ex situ XPS and further corroborated the transition-metal valence evolution using L_2,3_-edge spectroscopy.^10^ Similarly, ex situ XPS of P2–Na_0.76_Cu_0.22_Fe_0.30_Mn_0.48_O_2_ demonstrated reversible Cu^2+^/Cu^3+^ and Fe^3+^/Fe^4+^ evolution during Na^+^ extraction/insertion, with Fe redox contributing substantially to the high-voltage charge compensation.^46^ Operando XAS studies on Cu–Fe–Mn P2 oxides further establish the sequential participation of transition-metal redox during Na^+^ extraction.^47^ In addition, systematic Cu substitution studies in P2 Mn-based oxides have demonstrated the electrochemical accessibility of Cu redox, while recent work on Na_0.67_Fe_0.3_Mn_0.5_Cu_0.2_O_2_ showed that Cu incorporation can enhance the high-voltage Fe^3+^/Fe^4+^ redox activity.^48^ Collectively, these results establish a strong literature basis for considering Cu^2+^/Cu^3+^ and Fe^3+^/Fe^4+^ as complementary charge-compensation processes in NCFM10.

Ex situ XRD analysis of NCFM10 (Figure 7a) reveals distinct structural evolution during sodium (de)­intercalation. Upon charging, the (002) reflection exhibits a pronounced shift toward lower 2θ values, indicative of interlayer expansion driven by Na^+^ extraction and the resulting enhancement in electrostatic repulsion between adjacent oxide slabs.^49^ This shift is a hallmark of layered oxide cathodes and reflects the tunability of interlayer spacing with sodium content. Upon discharge, the (002) peak reversibly shifts back toward its original position, signifying lattice re-expansion due to Na^+^ reinsertion. Notably, the discharged pattern shows reflections with comparable intensity and sharpness to the pristine state, confirming the high structural reversibility. The structural evolution of NCFM10 during Na^+^ extraction/insertion was quantitatively evaluated from the ex situ XRD patterns by analyzing the characteristic peak positions and corresponding lattice parameters. In the pristine state, the hexagonal lattice exhibited *a* = 2.884 Å and *c* = 11.163 Å, corresponding to a unit-cell volume of 80.41 Å^3^. Upon charging, the a and c parameters increased slightly to 2.888 Å and 11.419 Å, respectively, resulting in a unit-cell volume of 82.480 Å^3^, corresponding to a modest volume expansion of 2.57%. After discharge, the lattice parameters changed to *a* = 2.898 Å and *c* = 11.120 Å, giving a unit-cell volume of 80.87 Å^3^. Thus, the discharged state retained approximately 99.42% of the pristine unit-cell volume, with only a 0.58% difference from the initial state (Table S2). These limited lattice changes and substantial recovery of the unit-cell volume demonstrate the good structural reversibility and stability of the NCFM10 framework during Na^+^ cycling. Figure 7b provides a magnified view of the (002) reflection, further highlighting the reversible contraction and expansion of the lattice. Figure 7c shows the corresponding galvanostatic charge–discharge profile of NCFM10 at 0.1C within the voltage range of 2.2–4.2 V, demonstrating reversible Na^+^ extraction and insertion. Overall, the ex situ XRD findings affirm that NCFM10 retains the P2-type layered structure during cycling, indicating the stability of the material.

**Figure 7:**
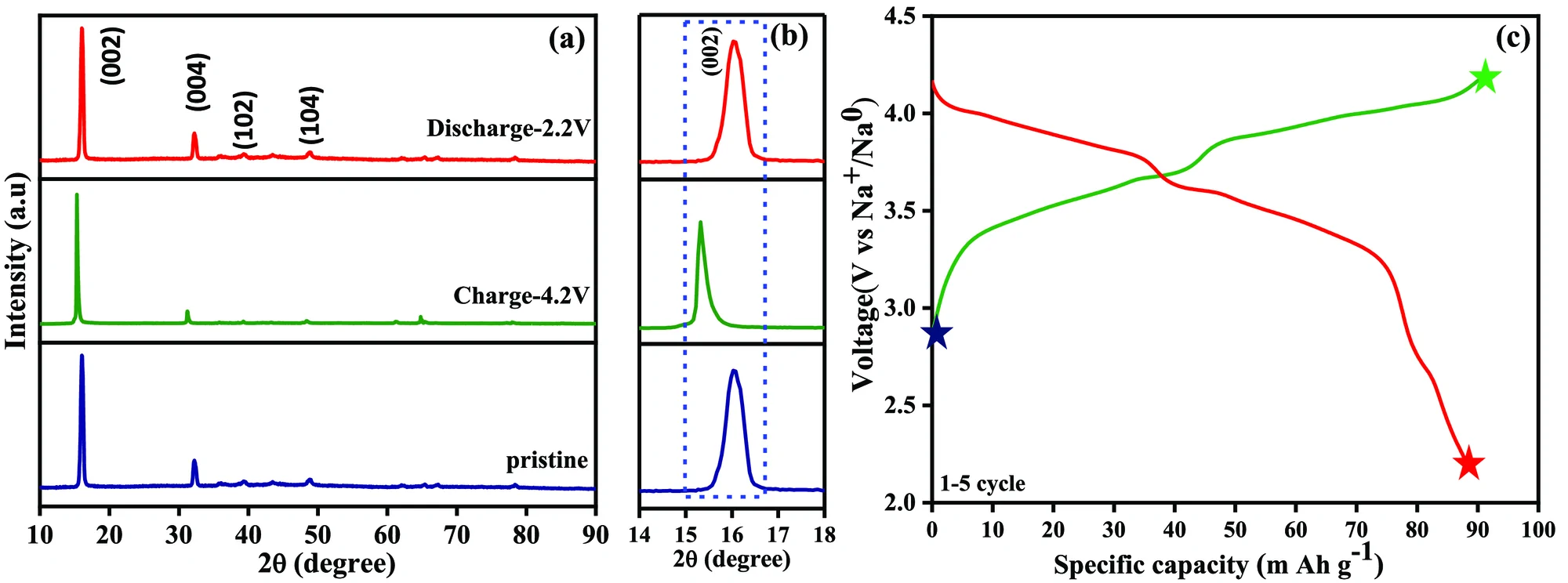
(a) Ex situ X-ray diffraction (XRD) patterns of NCFM10 in pristine, charged, and discharged states. (b) Magnified view of the ex situ XRD patterns. (c) The corresponding galvanostatic charge–discharge profile of NCFM10 at 0.1C.

## 4 Conclusion

In conclusion, P2-type layered oxides with the nominal composition Na_0.7_Cu_0.3_Fe_0.1_Mn_0.6_O_2_ were successfully synthesized via a citric acid-assisted sol–gel method with varying excess sodium contents. Among these, the composition prepared with 10 wt % excess Na (NCFM10) exhibited superior electrochemical performance, demonstrating enhanced capacity, stability, and high average operating voltage of ∼3.54 V. To further optimize conductivity, carbon nanotubes (CNTs) were incorporated into NCFM10, resulting in significantly improved charge transport, reduced polarization, and a stabilized electrode–electrolyte interface. The optimized NCFM10-CNT composite delivered the highest initial capacity (∼90 mAh g^–1^) with excellent capacity retention (∼94% after 100 cycles) and nearly 100% Coulombic efficiency. Phase transitions and structural evolution during cycling were investigated by ex situ XRD, confirming the absence of irreversible phase transitions. In addition, X-ray photoelectron spectroscopy (XPS) demonstrated the active participation of Cu redox processes in the electrochemical mechanism. This robust cycling stability and high-rate capability underscore the synergistic effect of sodium-excess tuning and CNT incorporation, offering a promising strategy for the design of high-performance sodium-ion battery cathodes. The enhanced electrochemical performance can be attributed to the synergistic interaction between Cu and Fe redox couples within the layered framework. While Cu^2+^/Cu^3+^ maintains high-voltage operation, Fe^3+^/Fe^4+^ extends cationic redox participation and reinforces structural stability by reducing Jahn–Teller distortion through the oxidation of Mn^3+^ to Mn^4+^. This cooperative redox behavior ensures reversible Na^+^ insertion and extraction, leading to improved cycling stability and overall electrode integrity. In summary, the observed performance trend across the studied compositions indicates that controlled sodium excess and Fe substitution play a supportive role in stabilizing the layered structure and maintaining electrochemical reversibility. The combined structural and electrochemical observations consistently show reduced polarization and stable cycling response for the optimized composition, reinforcing that compositional tuning and conductive network modification are practical approaches for improving P2-type Cu–Mn-based cathode systems.

## Supplementary Material



## Notes

Additional morphological and elemental characterization, including FESEM-EDS elemental mapping of NCFM0, NCFM5, and NCFM10; XPS characterization, electrochemical characterization, including galvanostatic charge–discharge profiles, differential capacity (d*Q*/d*V*) curves, and cycling performance of NCFM0, NCFM5, and NCFM10 with different carbon additives; ex situ XPS analysis of NCFM10 electrodes at pristine, charged, and discharged states; lattice parameters of NCFM cathodes with different sodium contents; and lattice parameters of NCFM10 obtained from ex situ XRD at different electrochemical states (Figures S1–S6 and Tables S1 and S2) (PDF)
