# Supplementary material for: Tuning High-Voltage Cu–Fe Redox in P2-Type Layered Metal Oxide Cathodes for Sodium-Ion Batteries
Source: ACS Omega. 2026 Oct 1;11(40):62115–25. doi: 10.1021/acsomega.6c08581 (PMC13670807; doi:10.1021/acsomega.6c08581)
Supplement: Supplementary file 1 [file ao6c08581_si_001.pdf]

Supporting Information

Tuning high voltage Cu-Fe Redox in P2-type layered metal oxide cathodes for Sodium-Ion Batteries

*Hina Parappan, Greeshma Caroline, Shantikumar V. Nair, Senthilkumar Baskar**

School of Nano sciences and Molecular Medicine, Amrita Vishwa Vidyapeetham, Kochi, Kerala 682041, India

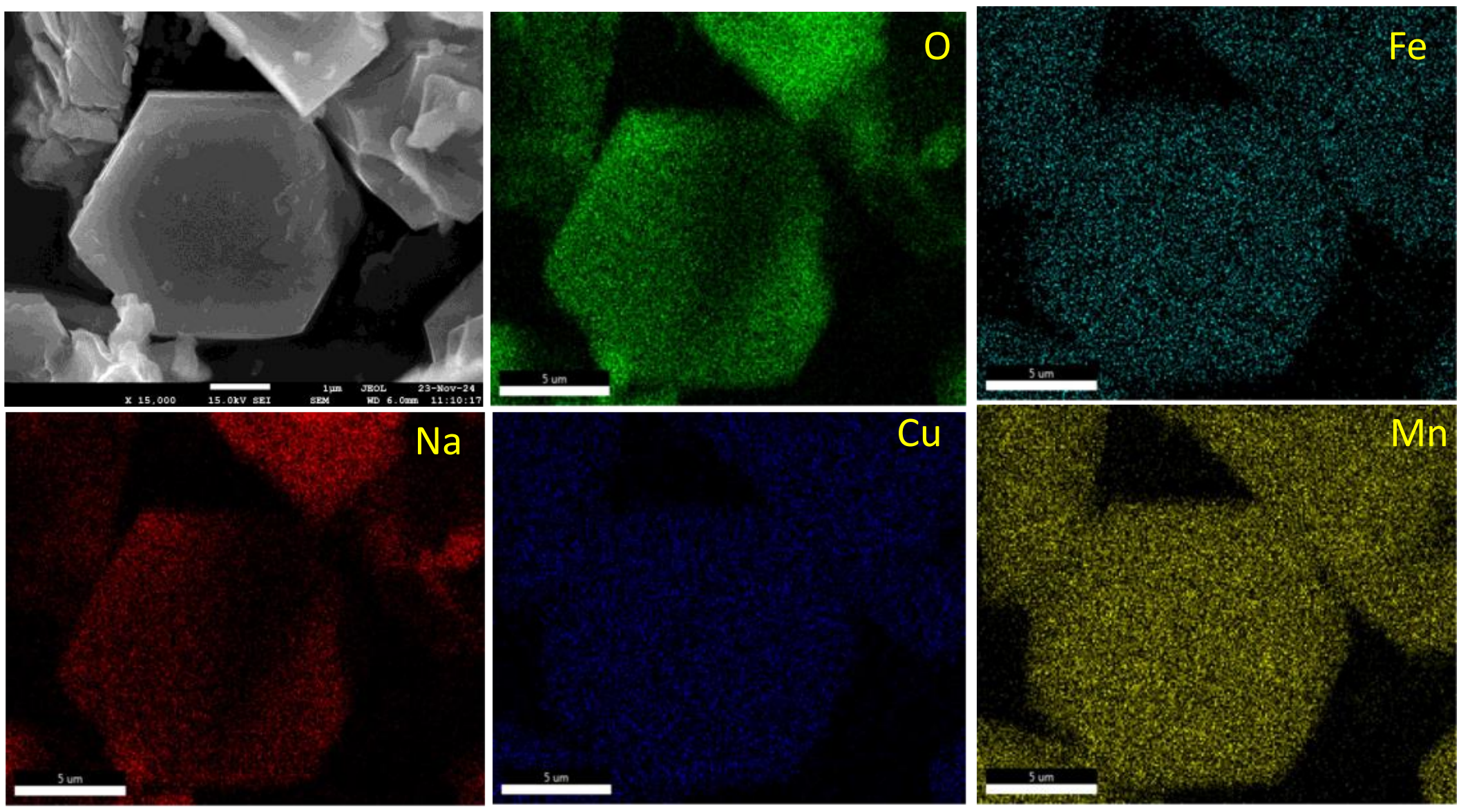

Figure S1

FESEM-EDS elemental mapping of NCFM0 reveals a homogeneous elemental distribution, confirming the successful and uniform incorporation of all constituent elements

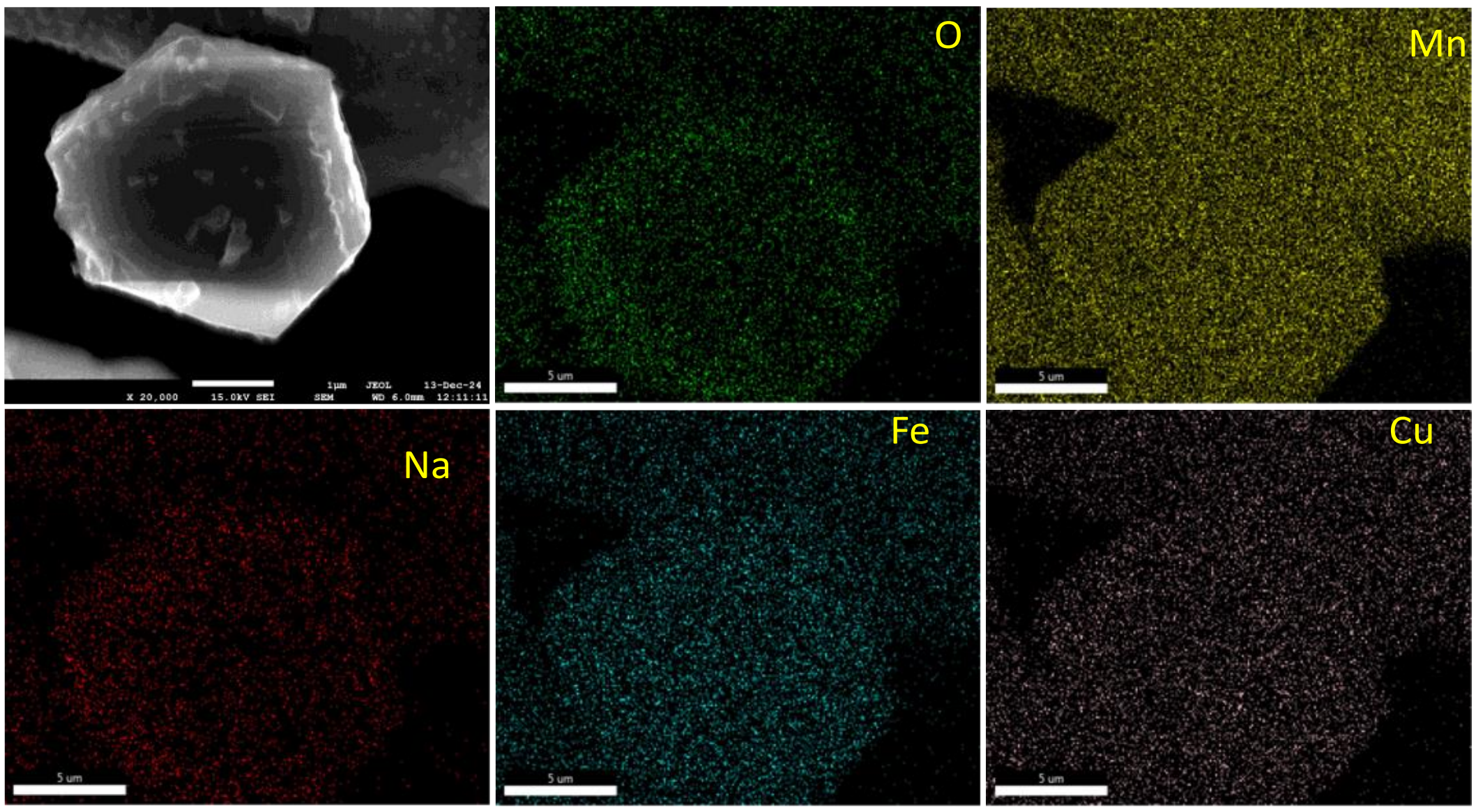

Figure S2

FESEM-EDS elemental mapping of NCFM5 reveals a homogeneous elemental distribution, confirming the successful and uniform incorporation of all constituent elements.

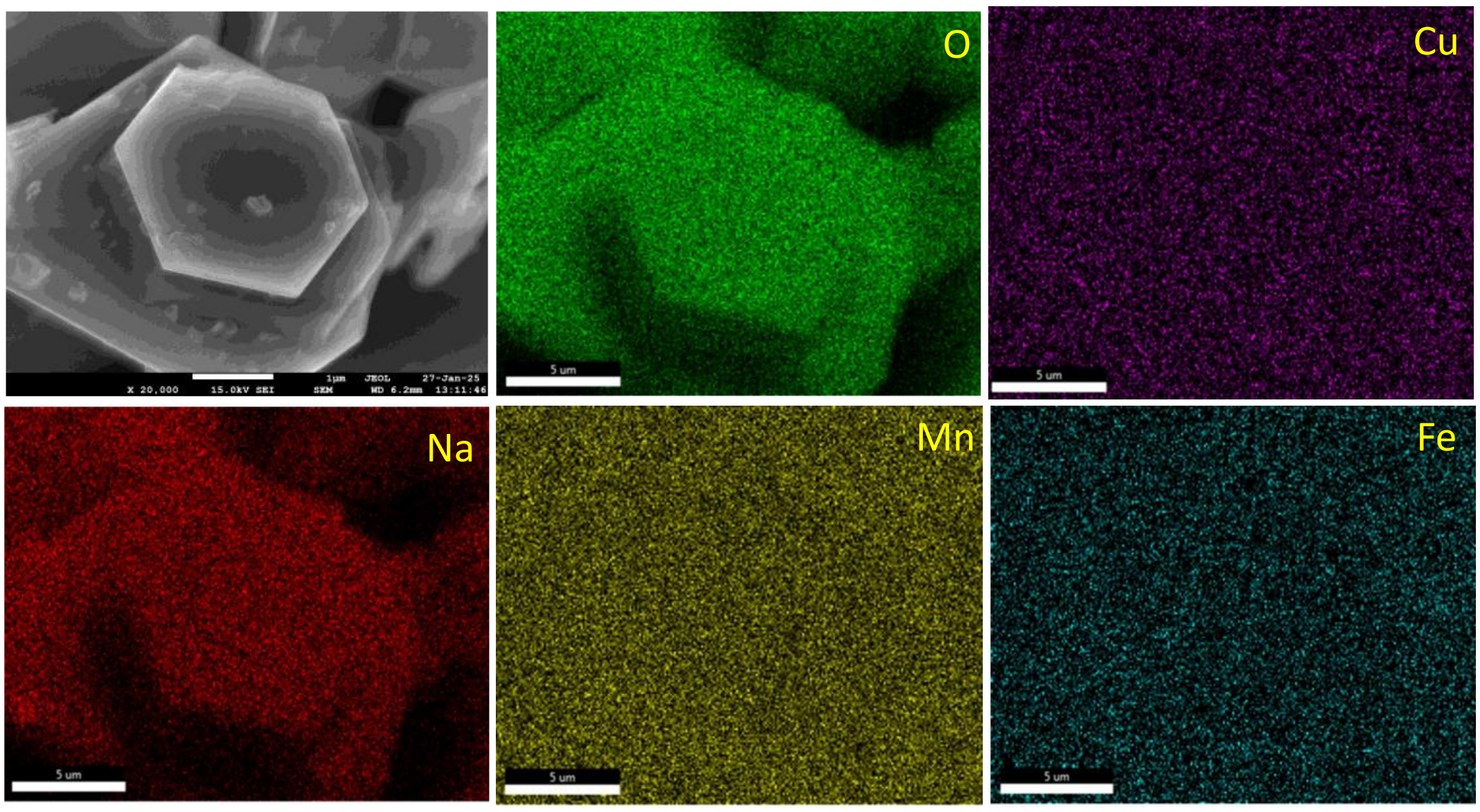

Figure S3

FESEM-EDS elemental mapping of NCFM10 reveals a homogeneous elemental distribution, confirming the successful and uniform incorporation of all constituent elements.

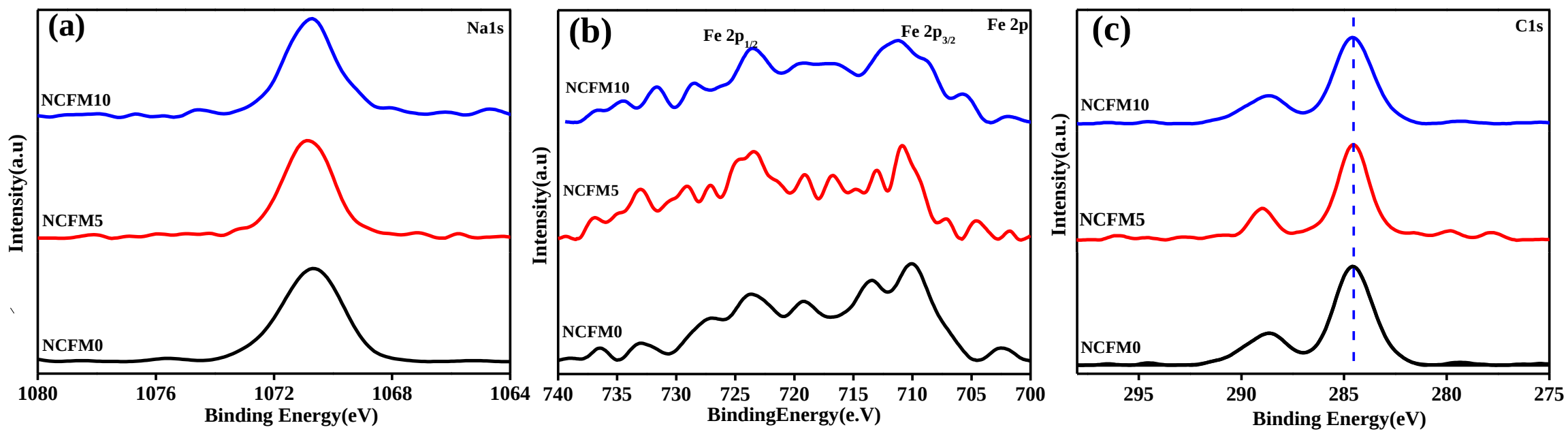

Figure S4

X-ray photoelectron spectroscopy (XPS) spectra of the materials NCFM0, NCFM5, and NCFM10, illustrating the elemental composition and chemical states. (a) Na 1s spectrum. (b) Fe 2p spectrum. (c) C 1s spectrum

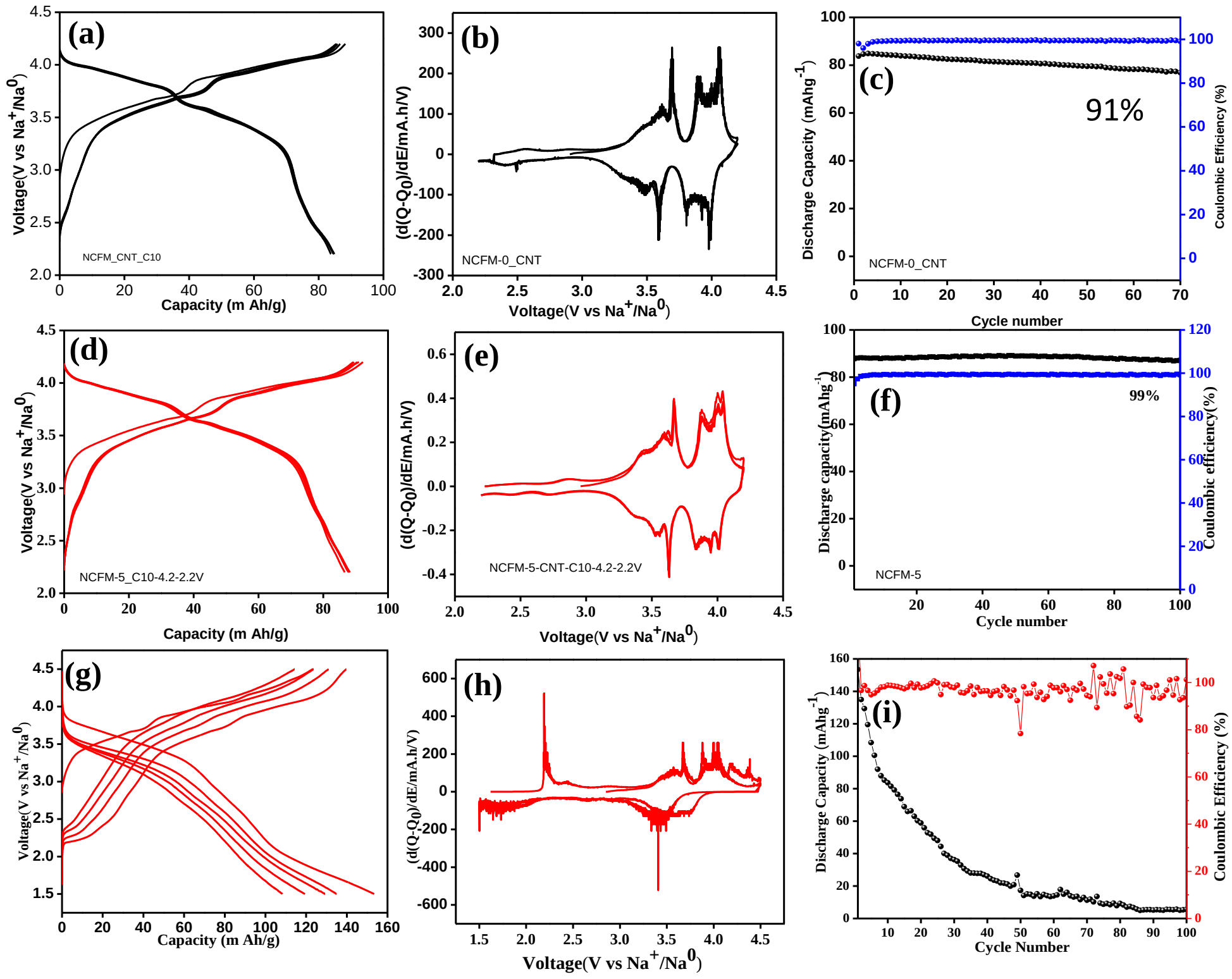

Figure S5. (**a**) Galvanostatic charge–discharge profiles of CNT additive of NCFM0 samples at 0.1C over the voltage range 4.2–2.2 V. (**b**) corresponding Differential capacity (dQ/dV) profiles within the same voltage window. (**c**) Cycling performance at 0.1C.(**d**) Galvanostatic charge–discharge profiles of CNT additive of NCFM5 samples at 0.1C over the voltage range 4.2–2.2 V. (**e**) corresponding Differential capacity (dQ/dV) profiles within the same voltage window. (**f**) Cycling performance at 0.1C.(**g**) Galvanostatic charge–discharge profiles of Carbon black additive of NCFM10 samples at 0.05C over the voltage range 4.5–1.5V. (**h**) corresponding Differential capacity (dQ/dV) profiles within the same voltage window. (**i**) Cycling performance at 0.05C.

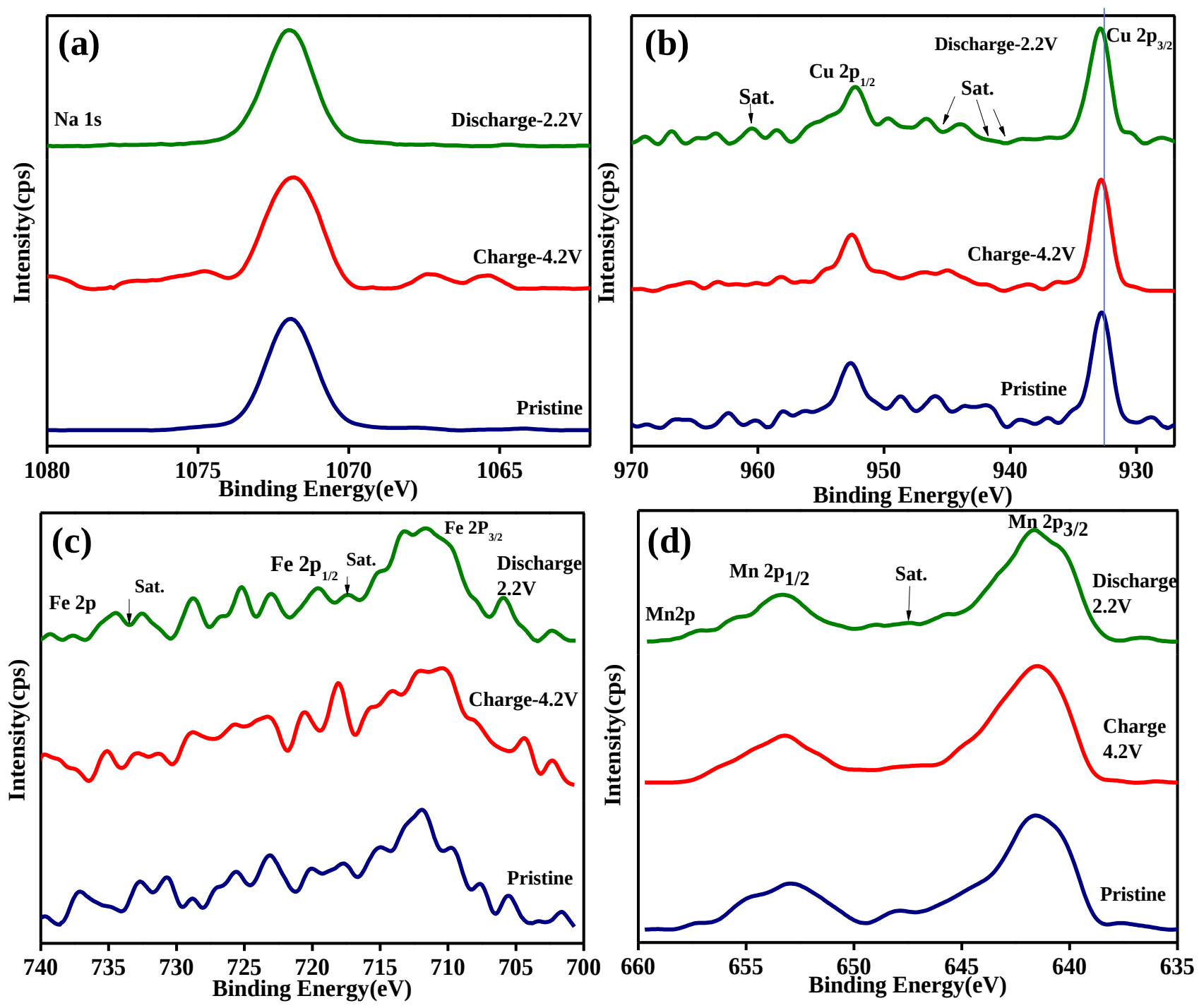

Figure S6. Ex -situ X-ray photoelectron spectroscopy (XPS) spectra of the elements (a) Na 1s, (b) Cu2p (c) Fe 2p and (d) Mn2p in NCFM10 electrode at pristine, charged and discharged conditions.

Table S1

Lattice parameters of the sample $Na_{0.7}Cu_{0.3}Fe_{0.1}Mn_{0.6}O_2$ in various sodium weight percentage.

	NCFM0	NCFM5	NCFM10
Lattice			
Space group	*P6₃/mmc*	*P6₃/mmc*	*P6₃/mmc*
a(Å)	2.9035	2.9080	2.9080
b(Å)	2.9035	2.9080	2.9080
c(Å)	11.1632	11.1693	11.1693
v(Å³)	81.4982	81.7962	81.7962

Table S2

Lattice parameters of Ex-Situ XRD of NCFM10 at various condition.

**States**	**a (Å)**	**c (Å)**	**Unit-cell volume (Å³)**
Pristine	2.884	11.163	80.41
Charged	2.888	11.419	82.48
Discharged	2.898	11.120	80.87
